# The Slower Method the Truer One

**Published:** 1896-07

**Authors:** 


### EDITORIAL.

### THE SLOWER METHOD THE TRUER ONE.

With the rapid pace in all things the Americans have learned to go fast in one direction that is neither safe, wise, nor well. The inconsiderate creation of professorships and professors has been too rapid and untenable to longer continue, and the need of returning to the slower and truer methods, too forcibly facing us, to longer continue in the same dangerous pathway, must now be accepted as a duty to be religiously performed. The appointing of men without experience, training, or special equipment to professorships, when these honors should only cloak the shoulders of those who, by years of teaching, special preparation, and meritorious work of a high character, commanded them, has had its culmination in the hot-bed system of turning out from our veterinary colleges half-equipped, half-educated, untrained, and unsafe veterinarians to assume the responsibilities that fall to the lot of the practitioner in every community.

Honors that come so easily rest with little conception of the grave responsibilities such places and positions should carry in their train on the shoulders of those who wear them. The multiplication of veterinary colleges and the eagerness of local veterinarians to pose as professors for local celebrity and personal gain have been fruitful evils that have already cost us years of labor, of earnest thought, and solicitude to correct, and placed every association in the country on the defensive to shelter and protect their members from severe criticism and ridicule, because they were graduates of schools maintained on this basis. Censure that in most instances was well deserved. We therefore urge our readers to use their influence that this danger may not continue. Above all, let every man to whom the tempting allurement of a professorship is held forth consider well his own fitness for such a place. It is not to be determined upon the basis that he believes himself as well equipped or even better than some one he knows who fills a similar place in some college faculty. The true student, the earnest veterinarian of deep conviction, will fully realize how empty such an honor must be when once accepted, and will appreciate how great the duties, how grave the responsibilities which, when only half discharged, must bring ridicule and disappointment.

HALT THE DANGEROUS FARCE!

